# Investigating the Utility of Fractal Measures as a Diagnostic for Glaucoma

**DOI:** 10.1002/sim.70655

**Published:** 2026-08-18

**Authors:** Jonathan W. Henderson, Benjamin M. Davis, Hannah J. Mitchell

**Affiliations:** ^1^ School of Mathematics and Physics Queen's University Belfast Belfast UK; ^2^ UKRI‐STFC Central Laser Facility Rutherford Appleton Laboratory Didcot UK

**Keywords:** fractal, lacunarity, point patterns, succolarity

## Abstract

Recent advances in Adaptive‐Optics Confocal Scanning Laser Ophthalmoscopy (AO‐cSLO) have enabled non‐invasive, in‐vivo imaging of retinal cell subsets, at near single‐cell resolution. However, challenges such as spatial and temporal variability, limited observation windows, and segmentation errors hinder the effective use of this data for diagnosing neurodegenerative disorders like glaucoma. Common point pattern metrics used in this area, such as density and distance‐based measures, are sensitive to noise and incomplete data, reducing their diagnostic utility. Fractal measures, with their scale‐invariance and robustness to point pattern degradation, offer a promising alternative. In this study, we apply fractal analysis to RGC point pattern data sets from rodent glaucoma models and controls, comparing fractal dimension, lacunarity, and succolarity with traditional metrics. We find that lacunarity and succolarity provide more accurate and enable earlier detection of glaucoma than traditional metrics, demonstrating greater robustness to noisy and degraded data. These measures demonstrate significant potential for achieving an earlier and more reliable glaucoma diagnosis.

## 1 Introduction

Glaucoma is a neurodegenerative disease and leading cause of irreversible blindness worldwide. It is projected that approximately 111 million people will be affected globally by 2040 [1]. The disease is characterized by morphological changes in the optic nerve head, resulting in the loss of retinal ganglion cells (RGCs), which are responsible for transmitting visual information from the eye to the brain [1]. Previous studies have identified various risk factors, including the patient's age, race, and family history [2]. However, there is currently no cure for glaucoma, and existing treatments are most effective in the early stages of the disease. Therefore, an early diagnosis is crucial, as it maximizes the window for effective therapeutic intervention to slow disease progression [3]. However, several challenges hinder the early diagnosis of glaucoma. One significant issue is the high plasticity of the central nervous system, which often compensates for early damage, causing patients to experience only subtle visual disturbances. These disturbances are typically insufficient to prompt noticeable symptoms until the disease has progressed to an advanced stage, delaying medical intervention unless routine diagnostic screening is performed [4]. Additionally, substantial variability in retinal cell populations and layer thicknesses exists across individuals, further complicated by age‐related degeneration. This heterogeneity makes it difficult to establish consistent and universal diagnostic criteria [5, 6]. Another complicating factor is inflammatory masking, where neurodegenerative retinal thinning is obscured by inflammation‐induced thickening, limiting the reliability of retinal layer measurements for tracking disease progression [7]. Moreover, retinal degeneration is not exclusive to glaucoma but is also implicated in other central nervous system disorders, such as Alzheimer's disease [8] and schizophrenia spectrum disorders [9], further complicating diagnosis. Differentiating diagnostic criteria for these conditions presents both a challenge and an opportunity for advancing diagnostic precision. Furthermore, the resolution of current structural imaging techniques, such as Optical Coherence Tomography (OCT), remains limited, which constrains the ability to detect subtle changes that may occur in the early stages of the disease [10], potentially requiring multiple visits to the clinic to establish progression. Nevertheless, recent advances in non‐invasive retinal imaging have made it possible to visualize human retinal cells at single‐cell resolution, including retinal ganglion cell (RGC) populations [11] and cells undergoing apoptosis [1]. Despite significant progress, these techniques are typically limited to visualizing a small subset of the entire RGC population. Consequently, the measurement processes are often noisy and prone to errors. To address these challenges, the present study utilizes rodent models of glaucomatous neurodegeneration to develop more robust methods for analyzing retinal point patterns. These approaches aim to enhance the accuracy and reliability of insights derived from high‐resolution imaging techniques.

In particular, we focus on using fractal measures to detect changes in the structure and density of RGCs. Previous studies have explored these measures to assess the complexity of RGC patterns [12, 13], with a particular focus on fractal dimension (FD). The FD is a widely used measure in the analysis of fractal point patterns, serving as an indicator of pattern complexity by assessing how pattern details change with the scale of measurement [14]. In the field of point pattern analysis, the FD finds diverse applications. For instance, Caballero et al. [15] proposed a test for complete spatial randomness based on the FD, offering an alternative to the commonly used distance‐based measures such as Ripley's K function and the F and G functions. Maanijou et al. [16], employed the FD to analyze the spatial distribution of Gold mineralization. The widespread utilization of the Fractal dimension is attributed to its simplicity in calculation, often employing the box‐counting algorithm [14]. Nonetheless, the algorithm has limitations as a measure of point pattern degradation. This is because calculation of FD necessitates only counting the number of boxes containing points and does not consider the number of points within each box, making this metric resilient to point pattern thinning. While this may be useful in situations where point‐pattern measurements are subject to noise, it also makes this metric potentially less‐sensitive to disease associated point pattern perturbations and therefore as a metric of disease progression. However, there are alternative and less well‐established fractal characterizations, namely, lacunarity [17] and succolarity [18], which are not subject to these limitations. In particular, lacunarity is calculated using a gliding box algorithm for its calculation, taking into account the density of points within boxes of varying sizes. By contrast, succolarity assesses percolation degree and considers the amount of empty space between points. These properties are less explored than fractal dimension. To the authors' knowledge, neither lacunarity nor Succolarity have been applied to the study of point patterns representing changes in cellular distribution in neurodegenerative disease models over time. This paper introduces tools for calculating lacunarity and succolarity and examines whether these fractal‐based point pattern measures provide greater diagnostic utility than traditional methods for detecting glaucoma‐related changes. We also explore the performance of these measures in the presence of noise, mimicking clinically derived, degraded RGC datasets. The results will highlight the robustness of these fractal measures, showing their potential to better handle noisy, clinically relevant data sets with the potential to improve early disease diagnosis.

## 2 Methods

### 2.1 Point Pattern Statistics

Previous studies for the detection of disease in retinas have focused on commonly used summary statistics from point pattern analysis [19]. In particular, nearest neighbor distance (NND), regularity index (RI), and average RGC size. The NND examines the distance between each point and the closest point to it. The distance is calculated using the Euclidean distance as shown in Equation (1) for two points (x,y) and (a,b) [20].

(1) $$d=\sqrt{{\left(x-a\right)}^{2}+{\left(y-b\right)}^{2}}.$$

Therefore, the average nearest neighbor can be calculated using Equation (2)

(2) $$X_{NND}=\frac{\sum_{i=1}^{N}d_{i}}{N},$$

where $d_{i}$ is the nearest neighbor distance for point i and N is the number of points.

Using the average NND, $X_{NND}$, the RI can be determined using Equation (3) [20].

(3) $$RI=\frac{X_{NND}}{\sum_{NND}},$$

where $\sum_{NND}$ is the standard deviation of the NND. The average RGC size is calculated by dividing the sum of the cell areas contained in the pattern by the total number of cells in the pattern.

### 2.2 Fractal Dimension

The most commonly used fractal characterization in point pattern statistics is the FD. FD can be calculated using a box counting algorithm [14]. Within this approach, the study region is covered in non‐overlapping squares with sides of length r. The number of boxes, N, that contain one or more points is counted and recorded. The procedure is then repeated, shrinking the size of the boxes for each subsequent calculation. The FD, D, is calculated as the slope of the line for the plot of $\log\left(\frac{1}{r}\right)$ against log(N). That is,

(4) $$D=\frac{\log(N)}{\log\left(\frac{1}{r}\right)}.$$

The resulting value of the fractal dimension will lie between 0 and the Euclidean dimension of the embedding space.

### 2.3 Lacunarity

Lacunarity is defined as a measure of the gap distribution [18]. If a fractal has a large value for the lacunarity, it implies that there is more space between points in the fractal. The calculation of lacunarity can be achieved by using a sliding box algorithm as suggested by Plotnick et al. [18]. In this algorithm, a box of length r is placed at the origin of the study region. The number of points contained within the box is counted. The box is then shifted along a space in the study region, and the number of points within the box are again determined. This is repeated for the entire study region and therefore produces a frequency distribution of the box masses n(s,r). Dividing the frequency distribution by the total number of boxes of size r, N(r), produces the probability distribution Q(s,r). Therefore, the first and second moments of the distribution are given as:

(5) z(1)=∑sQ(s,r),

(6) $$z(2)=\sum s^{2}Q(s,r).$$

The lacunarity can then be calculated as follows:

(7) $$\Lambda =z(2)/{\left[z(1)\right]}^{2}.$$

The calculation can then be repeated for various box sizes r. As a result, a log‐log of the lacunarity versus the size of the box can be produced for comparison between patterns.

### 2.4 Succolarity

Succolarity can be thought of as indicating the capacity of a flow to cross the study region [17]. Four directions are considered in the calculation of succolarity. They are top to bottom (T2B), bottom to top (B2T), left to right (L2R), and right to left (R2L). Furthermore, in addition to the fluid direction, a pressure field is also considered where the pressure field grows from left to right and top to bottom. The algorithm to analyze the succolarity is as follows:

*Step 1*. Convert the point pattern to a binary image. Empty space is given a value of 0 and points are given a value of 1. Areas with a value of 0 are the flood areas as fluid can pass through them without obstruction.
*Step 2*. Construct four new images by considering the path that a fluid travels from each of the four directions. That is, T2B, B2T, R2L, and L2R. Areas which fluid can pass through without obstruction are given a value of 1, whilst areas which it cannot pass through are given a value of 0.
*Step 3*. Overlay the resulting images with boxes of size k×k pixels.
*Step 4*. For each box, calculate the occupation percentage. That is, the fraction of pixels within each box that have a value of 1. For a box of size k×k pixels, this is denoted OP(BS(k)).
*Step 5*. A pressure field is applied to each image. The pressure increases from top to bottom for the two images T2B and B2T. For the remaining two images, the pressure field increases from left to right. For each box, the value of the pressure is taken from the corresponding x or y value of the centroid of the box. The pressure of the box of size k×k pixels is denoted PR(BS(k),pc), where *pc* denotes the x or y value of the centroid of the box.
*Step 6*. For a pixel image that has been divided into n equally sized boxes, the succolarity is calculated as follows:
(8) $$\sigma =\frac{\sum_{i=1}^{n}OPBS(k)\times PR(BS(k),pc)}{\sum_{i=1}^{n}PR(BS(k),pc)}.$$

**FIGURE 1:**
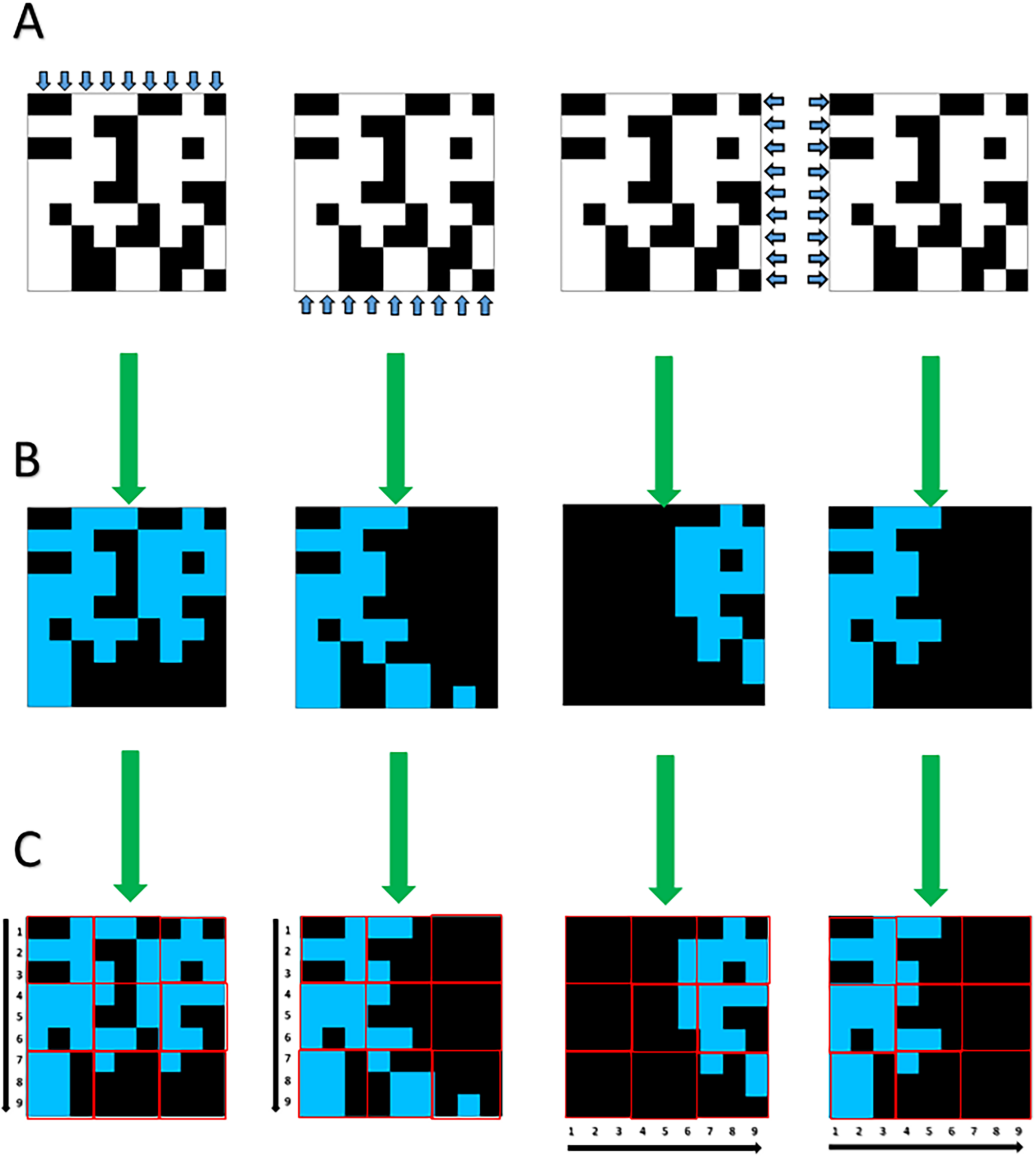
Succolarity algorithm steps. (A) The direction of travel of a fluid when applied to each of the four sides of the study region, that is T2B, B2T, R2L, L2R. The resulting images are shown in (B). The images are then superimposed with boxes of size 3×3 pixels, from which the occupation percentage can be calculated, and a pressure field is applied increasing from top to bottom for the T2B and B2T images and from left to right for the R2L and L2R images (C).

The above steps are summarized in Figure 1.

The value of the succolarity ranges from 0 to 1. A value close to 1 indicates that the fluid can pass through with little or no obstruction, whilst a value of 0 implies that the path the fluid travels before being obstructed is short. The selection of box size and number of pixels is discussed in the Appendix B.

## 3 Simulation Study: Evaluating Metric Sensitivity to Degradation and Noise

One of the challenges in analyzing the spatial distribution of RGCs from clinical retinal images is the presence of both degeneration and various forms of noise. Previous work, such as Davis et al. [19], identified the average nearest‐neighbor distance (NND) as a useful metric for detecting structural changes in RGC mosaics. However, their study did not assess the robustness of the metrics they used to different types of noise or gradual degradation.

A simulation study was used to evaluate the robustness and sensitivity of four metrics, NND, FD, lacunarity, and succolarity, to both point degradation and two types of noise: (1) positional perturbations and (2) false positive/false negative points sampled from CSR.

### 3.1 Simulation Setup

We generated 100 point patterns with approximately 300 points to represent healthy retinal ganglion cell (RGC) distributions. Three types of spatial patterns were considered:

- A *homogeneous Poisson process*
- A *clustered pattern*, generated using the Matérn Cluster Process
- A *regular pattern*, generated using the Simple Sequential Inhibition process

Table 1 gives an overview of the simulations that have been conducted including the levels of degradation, noise, false positives and false negatives and combined effects. 100 independent point patterns (of each of the three spatial types) were simulated for each level of effect.

**TABLE 1:** Simulation study overview.

Type of effect	Level range	Step size
Degradation	0.01–0.2	0.01
Noise	0, 0.01, 0.05, 0.1, 0.2, 0.3, 0.4	
False positive	0, 0.01, 0.05, 0.1, 0.2, 0.3, 0.4	
False negative	0,0.05,0.1,0.3	
Combined effect		
Degradation, noise, false positive, false negative	All combination of levels defined	

### 3.2 Diagnostic Discriminability Using ROC Analysis

To quantify the ability of the spatial metrics to distinguish between control and diseased conditions under simulated perturbations, receiver operating characteristic (ROC) analysis was performed using the pROC package [21] in R.

For each simulation condition (baseline, degraded patterns, and noise‐perturbed patterns), metric values derived from control patterns were treated as the negative class, while values derived from diseased or altered patterns were treated as the positive class. ROC curves were constructed by varying the classification threshold across the full range of observed metric values and computing the corresponding sensitivity and specificity at each threshold.

The area under the ROC curve (AUC) was used as a summary measure of discriminatory performance. An AUC value of 0.5 indicates no discriminatory ability, whereas values closer to 1 indicate stronger separation between classes.

ROC analysis was applied consistently across both degradation and positional noise experiments to assess how metric separability changes under increasing levels of simulated impairment. In the degradation analysis, AUC values were computed at each level of point removal, while in the positional noise analysis, AUC values were computed across increasing levels of Gaussian perturbation.

As this analysis is embedded within a simulation framework, ROC performance reflects the robustness of metric separability under controlled perturbations rather than clinical classification performance. No train‐test split or cross‐validation was performed, as the objective was not predictive modelling but evaluation of metric stability under known simulated conditions.

It should also be noted that multiple observations may be derived from related simulated point patterns, and therefore independence between samples may be limited. As such, ROC results should be interpreted as measures of discriminatory signal strength rather than absolute estimates of clinical diagnostic accuracy.

### 3.3 Sensitivity to Degradation

To simulate neurodegeneration, we systematically degraded the point patterns by randomly removing a proportion of points. Degradation levels ranged from 0.01 to 0.2, in increments of 0.01. For each level, 100 independent simulations were conducted. Each metric was assessed for its ability to detect statistically significant changes from the baseline pattern.

Table 2 summarizes the minimum degradation level at which each metric consistently detected a significant change (defined as achieving statistical significance in at least 95 out of 100 simulations).

**TABLE 2:** Minimum degradation level at which significant changes were detected by each metric.

Metric	Random pattern	Clusteredpattern	Inhibitionpattern
Lacunarity	0.03	0.02	0.02
Succolarity	0.02	0.02	0.02
Fractal dimension (FD)	Not significant	Not significant	0.52
Nearest‐neighbour distance (NND)	0.09	0.1	0.08

The results show that the fractal measures of lacunarity and succolarity performed best, with detection occurring at degradation levels as low as 0.02–0.03 across all pattern types. In contrast, fractal dimension (FD) performed comparatively poorly, potentially due to the limitations of the box‐counting algorithm, which is not sensitive to subtle changes in local pattern density. NND was able to detect change but at larger degradation levels than lacunarity and succolarity.

### 3.4 Sensitivity to Positional Noise

To test metric robustness to noise in point locations (positional jitter), Gaussian noise was introduced by perturbing each point's x and y coordinates using $\epsilon_{x},\epsilon_{y}∼𝒩(0,\sigma^{2})$. The following noise levels were applied, reflecting increasing variance in point displacement: σ=0.01,0.05,0.1,0.2,0.3,0.4.

**FIGURE 2:**
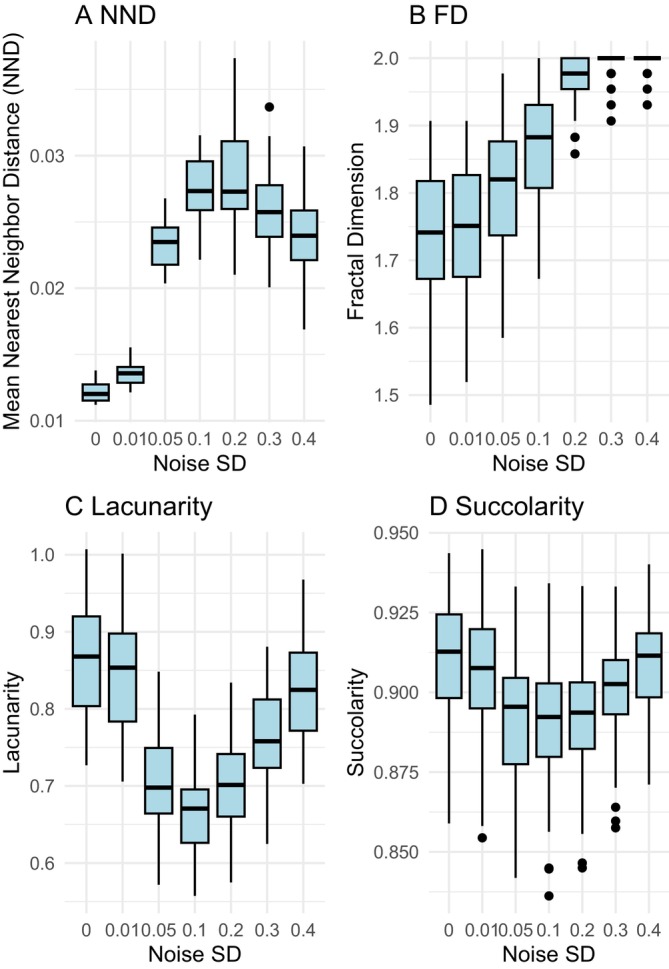
Effect of positional Gaussian noise on metric values for homogeneous Poisson point patterns.

Figure 2 shows the results of the simulation for point patterns generated using a homogeneous Poisson process. The Noise SD=0 is the noise‐free pattern.

As expected the NND metric was particularly sensitive to increasing levels of positional jitter, with a significant difference observed between the noise‐free pattern (Noise SD=0) and σ=0.05. In contrast, the fractal‐based metrics (lacunarity and succolarity) remained comparatively stable, showing some variation but no significant differences detected across all noise levels. The fractal dimension (FD) showed a significant change only at σ=0.3.

Similar trends were observed for clustered and regular point patterns (see appendix). Lacunarity and succolarity again showed no significant changes across noise levels, while NND detected significant differences at σ=0.05 for clustered patterns and at σ=0.1 for regular patterns. For FD, no significant difference was observed in regular patterns, while significance was detected at σ=0.4 for clustered patterns (see Appendix A).

### 3.5 Sensitivity to False Positive and False Negative Points

**FIGURE 3:**
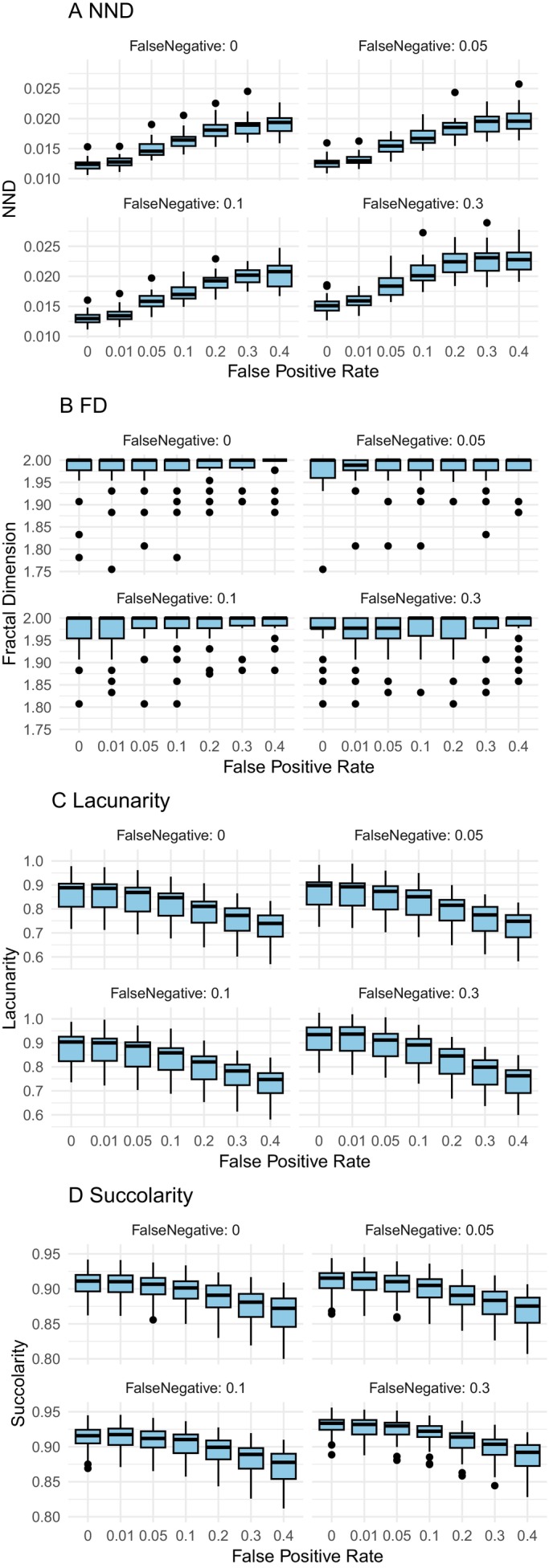
Effect of false positives/negatives on metric values for homogeneous Poisson point patterns.

In addition to positional jitter, we tested how robust the metrics were to false positive and false negative detections, mimicking segmentation errors or errors in the imaging process. False positives were added as random extra points, while false negatives were modelled by removing a random subset of existing points, without altering the spatial distribution. Figure 3 shows the results from the simulation applied to the homogeneous Poisson process for different levels of false positives and negatives.

The control pattern (noise‐free) corresponds to a scenario with zero false negatives and zero false positives. The NND metric detected a significant difference between the original pattern and patterns with a false negative rate of 0.3, across all false positive rates, whereas the fractal measures (FD, lacunarity, succolarity) showed no significant change. The same conclusions held for clustered and inhibition patterns: NND was consistently sensitive to noise, while FD, lacunarity, and succolarity remained unaffected across all tested noise levels (see Appendix A).

### 3.6 Combined Effects and Summary

We looked at the effects of combining both types of noise alongside degradation. In Tables 3, 4, 5, 6, the results of different combined noise types are analyzed and degradation is introduced at levels of 0.05 until 0.8 in increments of 0.05 to understand when each metric could detect significant changes to the point pattern when noise is present.

**TABLE 3:** Minimum degradation level at which significant changes were detected by NND whilst noise level was present.

Noise level (positional sd, false positive,false negative)	Randompattern	Clusteredpattern	Inhibitionpattern
(0.1, 0.1, 0.1)	0.65	0.6	0.6
(0.2, 0.2, 0.2)	0.65	0.55	0.6
(0.3, 0.3, 0.3)	0.6	0.5	0.65
(0.4, 0.4, 0.4)	0.65	0.55	0.65

**TABLE 4:** Minimum degradation level at which significant changes were detected by FD whilst noise level was present.

Noise level (positional sd, false positive,false negative)	Randompattern	Clusteredpattern	Inhibitionpattern
(0.1, 0.1, 0.1)	Not significant	Not significant	Not significant
(0.2, 0.2, 0.2)	Not significant	0.8	Not significant
(0.3, 0.3, 0.3)	Not significant	0.75	0.75
(0.4, 0.4, 0.4)	Not significant	0.75	0.75

**TABLE 5:** Minimum degradation level at which significant changes were detected by lacunarity whilst noise level was present.

Noise level (positional sd, false positive,false negative)	Randompattern	Clusteredpattern	Inhibitionpattern
(0.1, 0.1, 0.1)	0.15	0.2	0.25
(0.2, 0.2, 0.2)	0.15	0.15	0.25
(0.3, 0.3, 0.3)	0.2	0.15	0.25
(0.4, 0.4, 0.4)	0.2	0.2	0.3

**TABLE 6:** Minimum degradation level at which significant changes were detected by succolarity whilst noise level was present.

Noise Level (positional sd, false positive,false negative)	Randompattern	Clusteredpattern	Inhibitionpattern
(0.1, 0.1, 0.1)	0.1	0.1	0.15
(0.2, 0.2, 0.2)	0.15	0.1	0.2
(0.3, 0.3, 0.3)	0.2	0.15	0.2
(0.4, 0.4, 0.4)	0.2	0.15	0.25

When analyzing all forms of noise and degradation together, the results were consistent with those observed in previous sections. All metrics—except for the fractal dimension—were able to detect changes caused by structural degradation. The limited effectiveness of the fractal dimension is attributed to the nature of its calculation. In contrast, the fractal‐based metrics of lacunarity and succolarity demonstrated the most consistent and robust performance across all noise levels, detecting changes at relatively low degradation thresholds. Although the NND metric identified statistically significant changes, these were less prominent compared to those detected by lacunarity and succolarity. Furthermore, NND exhibited greater variability in both sensitivity and reliability under noisy conditions.

### 3.7 Simulation Discussion

Overall, succolarity and lacunarity consistently outperformed both NND and fractal dimension (FD). These metrics were robust to various forms of noises (including positional perturbations and false point insertions) while still being sensitive to degradation. Although NND was effective under ideal conditions, its high sensitivity to noise makes it less suitable for real‐world applications.

## 4 A Case Study

### 4.1 Data Set

**FIGURE 4:**
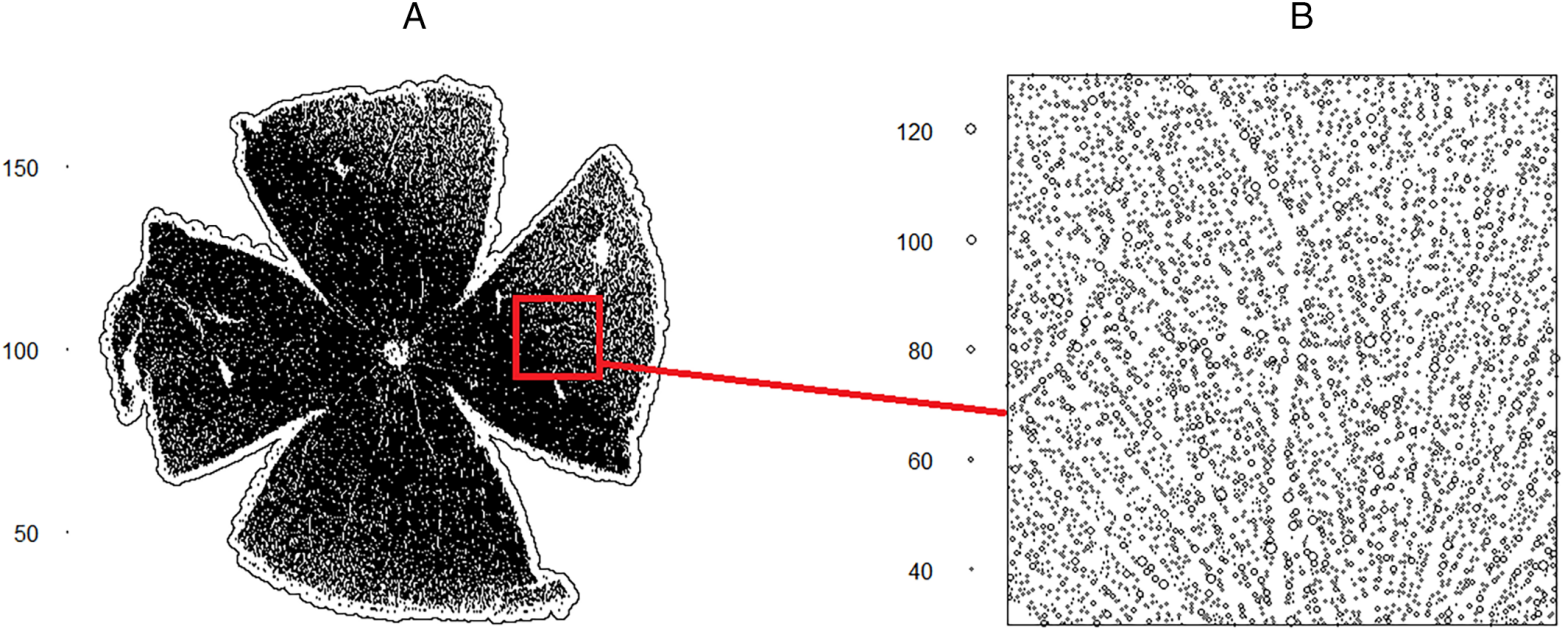
(a) A murine retinal RGC point pattern. (b) subsample of RGC point pattern, circle size depends on cell size.

Rodent models of the neurodegenerative processes underpinning Glaucoma have made a substantial contribution to the understanding of disease mechanism and help progress the development of new therapies [22]. Substantial point pattern data sets comprising over 2.5 million RGC positions and morphologies from ex‐vivo retinal whole‐mounts of experimentalglaucoma models, including the Morrisons Ocular Hypertension model (OHT) and Partial Optic Nerve Transection model (pONT), have previously been made available to investigate the spatial‐temporal properties of Glaucomatous RGC loss [11]. Here, RGC locations are identified through labelling with the nuclear restricted transcription factor Brn3a (see Figure 4 [11, 19, 23]).

The dataset used consists of two models of glaucoma disease with images of the flat‐mounted retina's taken in snapshots of time (day of dissection). Table 7 gives an overview of the number of whole‐mounts per time per disease model. Each of these retina were divided into study regions of size 300 × 300 *μ*m from distances 300 to 240 *μ*m from the optic nerve and treated independently.

**TABLE 7:** Glaucoma data for each diseased model retina.

Method	Time snapshot (day)	Number of retina
Healthy Control		15
Diseased		
pONT	3	4
pONT	7	6
pONT	21	9
pONT	56	5
OHT	7	5
OHT	21	9
OHT	56	4
OHT	84	4

The number and distribution of RGCs vary significantly within and between retinas in both human and rodents. As the number, distribution and normal rate of loss of RGCs due to aging can vary significantly and between individuals [24] this limits the diagnostic utility of density‐based measures of RGC population health in the absence of baseline measurements as establishing diagnostic relevant thresholds is non‐trivial. Viewing the RGC population as a point pattern allows for alternative approaches for the early detection of Glaucoma. Previously, using two well‐established rodent models of glaucoma, ocular hypertension (OHT), and partial optic nerve transection (pONT), Davis et al. [19] investigated the performance of measures from point pattern analysis when applied to small sub‐regions of the retina. These measures include the average nearest neighbour distance (NND), regularity index (RI), and average RGC size. The results of this study indicated that these measures performed poorly in detecting disease in the OHT model, which represents a milder model of RGC loss than pONT and is therefore used in this research to model early stages of the disease process. Considering the quality and scale of the histological DA‐rat glaucoma data set [11, 19, 20], this section explores the potential of fractal properties for early disease detection. As distance and density‐based measures have shown limitations in identifying early‐ stage disease [19], we investigate whether fractal characterizations of RGC point patterns offer a more effective diagnostic approach.

### 4.2 Bootstrapping Algorithm

In order to assess each of these measures diagnostic utility in identifying diseased and healthy populations, a bootstrapping procedure can be used as suggested by Davis et al. [19]. The procedure consists of the following steps,

1. A retina from the control group is randomly selected with replacement.
2. For each retina, a study region is selected at a corresponding distance from the ONH. The distances considered are from 300 to 2400. The study region at each distance is selected by generating a random set of coordinates corresponding to the center of the square. The size of this study region is chosen to be 300×300 as measures like succolarity, FD and NND require at least three points in a pattern to obtain a value so any pattern with less than three points is rejected but by setting the study region to this size means that at most 1% of patterns are being rejected within each group.
3. Due to the irregular shape of retinal wholemounts, the percentage of the study region contained within the retinal wholemount is calculated. If this value is less than 100%, the study region is rejected and the above steps are repeated until a suitable study region is found.
4. Once the region has been selected, the statistic of interest can be calculated (NND, RI, mean RGC area, FD, lacunarity, and succolarity) and the results recorded. For the succolarity, the number of pixels for the study region as well as the box size used also needs to be defined. In this project, the number of pixels was selected to be 50×50 so that the characteristics of the point pattern is captured. Furthermore, the succolarity value was obtained by taking an average for the four directions considered as they should be approximately equal as noted in de Melo et al. [17].
5. The process is repeated 1 000 00 times for the control group, generating 1 000 00 bootstrapderived metric observations per condition. The same procedure is then applied to the pONT and OHT groups, resulting in paired distributions of metric values for each comparison (control vs pONT and control vs OHT).
  For each comparison, ROC curves are constructed using the full set of bootstrap samples from both groups. Thus, each ROC/AUC curve is based on 100,000 control‐derived observations and 1 000 00 disease‐derived observations per condition. This large number of resampled observations provides a stable empirical approximation of the underlying metric distributions and supports robust estimation of the ROC curve.
  ROC analysis is implemented using the pROC package in R, [21] with classification thresholds varied across the empirical distributions of each metric. Performance is summarised using the area under the curve (AUC), as outlined in Section 3.2.
  To assess statistical reliability, uncertainty in AUC estimates is evaluated using bootstrap resampling inherent in the simulation framework. This allows confidence intervals for AUC to be derived directly from the empirical distribution of resampled values.
  Formal comparisons between metrics (e.g., differences in AUC between fractal dimension, lacunarity, and succolarity) are assessed using confidence interval overlap and paired comparisons across identical bootstrap iterations, ensuring that comparisons are performed on matched resamples rather than independent datasets.
  This approach ensures that ROC analysis is statistically meaningful within the context of the study design, as it is based on a large number of repeated independent resamples of retinal regions rather than single observations, allowing stable estimation of discriminatory performance under controlled sampling variability.

### 4.3 pONT: Glaucomatus RGC Loss in Rodent Model of Severe Disease

**FIGURE 5:**
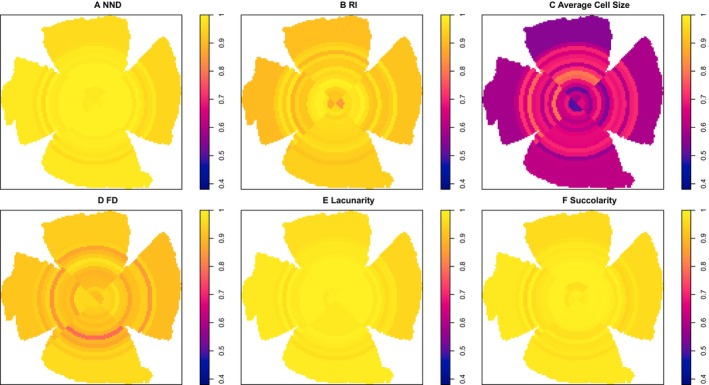
AUC values based on the distance from the ONH calculated from the pONT and control groups are illustrated for the (A) NND, (B) RI, (C) Average Cell Size, (D) FD, (E) Lacunarity, (F) Succolarity.

To evaluate the performance of fractal characterizations in comparison to standard measures, we applied the bootstrapping procedure [19] detailed above. Briefly, small square regions of interests were selected with replacement from the retina population in each condition corresponding to a region containing 300 RGCs (comparable to the AO‐cSLO field of view). For this analysis, all retina which had subject to the pONT model were pooled together for the bootstrapping procedure. The measures under investigation included those previously examined by Davis et al. [19], namely, Nearest Neighbor Distance (NND), Regularity Index (RI), and average cell size, along with the fractal measures introduced in this study: Fractal Dimension (FD), lacunarity, and succolarity. Each of these measures were calculated and comparisons were drawn between conditions through the construction of Receiver Operating Characteristic (ROC) curves to establish the potential diagnostic utility of each measure. To assess the effectiveness of each of these measures the Area Under the Curve (AUC) was calculated. Following Carter et al. classification [25], AUC values were categorized as follows: excellent for values between 0.9 and 1, very good for values between 0.8 and 0.9, adequate for values between 0.7 and 0.8, poor for values between 0.6 and 0.7, and failed for values less than 0.6. In Figure 5, plots are presented of the retina with corresponding AUC values at different distances from the Optic Nerve Head (ONH) when distinguishing between control retinas and retinas that underwent the pONT model, representing a more severe form of the disease.

The mean values of the AUC for each plot are summarized in Table 8 along with the 95% C.I.

**TABLE 8:** AUC values for comparison of pONT model and controls.

Method	Mean	95% C.I.
NND	0.98936	[0.98600, 0.99273]
RI	0.94797	[0.93998, 0.95595]
Average Cell Size	0.65063	[0.63352, 0.66774]
FD	0.91833	[0.90954, 0.92714]
Lacunarity	0.99122	[0.98813, 0.99430]
Succolarity	0.98559	[0.98192, 0.98886]

Except for the average cell size, all measures excel in distinguishing between healthy and diseased retinas subjected to the pONT model. The plots in Figure 5 reveal a consistent trend for each measure: samples taken closer to the Optic Nerve Head (ONH) exhibit higher AUC values than those taken further away. This implies a higher density of Retinal Ganglion Cells (RGCs) being lost in the central retina, near the ONH, resulting in increased space between cells compared to the peripheral regions of the retina. The poor performance of the average cell size can be attributed to the dynamic nature of RGCs in response to injury [11].

### 4.4 OHT: Glaucomatus RGC Loss in Rodent Model of Mild Disease

**FIGURE 6:**
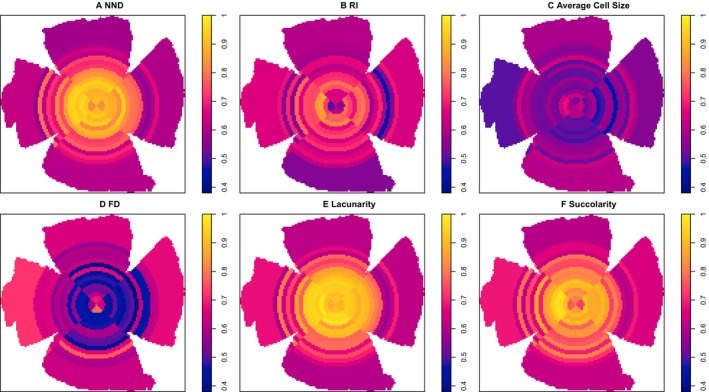
AUC values based on the distance form the ONH calculated from the OHT and control groups are illustrated for the (A) NND, (B) RI, (C) Average Cell Size, (D) FD, (E) Lacunarity, (F) Succolarity.

The bootstrapping procedure was repeated for both the control and OHT models. OHT is a milder form of the disease compared to pONT, making it suitable for evaluating the performance of each measurement method in early glaucoma detection. Figure 6 displays the AUC values at various distances from the ONH.

A summary of the average AUC values for each measure are shown in Table 9 along with the corresponding confidence interval.

**TABLE 9:** AUC values for the comparison of OHT model to controls.

Method	Mean	95% C.I.
NND	0.77920	[0.74732, 0.81108]
RI	0.67550	[0.65325, 0.69774]
Average Cell Size	0.55643	[0.54354, 0.56933]
FD	0.55228	[0.52645, 0.57811]
Lacunarity	0.80724	[0.77497, 0.83952]
Succolarity	0.78203	[0.75591,0.80815]

Comparing the OHT model, a milder form of glaucoma, to pONT, we observed lower AUC values than those presented in Table 6 and Figure 6. Among the conventional methods employed, the NND proved to be a reliable measure for disease detection, displaying a good performance (AUC values ≥0.8) when applied to samples from the central retina region. In contrast, the RI performed poorly in disease detection. This outcome suggests that while the NND appears suitable for detecting the disease in our study's samples, it may be sensitive to outliers, which can influence its efficacy. Furthermore, the average cell size yielded unsatisfactory results, largely due to its dynamic nature in response to injury, as previously mentioned. Additionally, the milder nature of the OHT model, compared to the pONT model, results in fewer RGC losses, leading to minimal changes in average cell size between healthy and diseased retinas in this model. The RI index, defined as the ratio of the average NND to the standard deviation of the NND, experiences an increase in both average NND and standard deviation due to RGC loss. This increase is a result of variations observed within each sample. When examining fractal measures, FD demonstrated poor sensitivity. The observed cell loss, particularly in the early stages of glaucoma, appears to occur randomly [11]. Since FD is calculated using the box‐counting algorithm, it only considers the presence of points within boxes, disregarding their density. However, both succolarity and lacunarity emerged as effective measures for distinguishing between diseased and healthy retinas, especially when applied to samples from the central retina region.

### 4.5 Examining the Performance of Distance and Fractal‐Based Measures for Early Diagnosis

**FIGURE 7:**
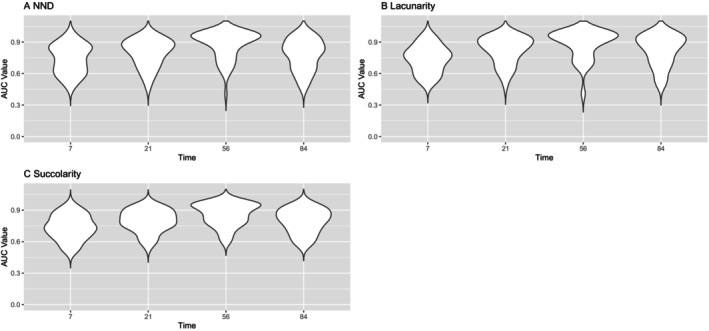
Violin plots showing the AUC values of (A) NND, (B) Lacunarity, and (C) Succolarity at different dissection times for the OHT model.

Among the six measures evaluated, lacunarity, succolarity, and NND emerged as the most accurate for distinguishing between diseased and healthy retinas, regardless of the severity of the disease. Given the importance of early glaucoma detection, this section investigates the performance of these measures over time. Focusing on the OHT model, particular attention is given to the central retina region, which is highly sensitive to disease‐related changes. In this analysis, retinal patterns are categorized based on the timing of retinal dissection. Figure 7 presents AUC plots for each measure at different dissection time points.

**FIGURE 8:**
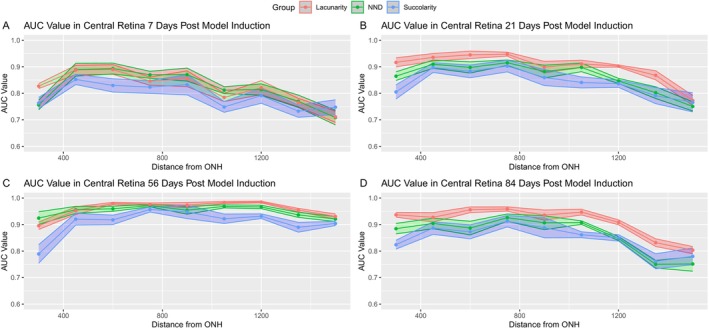
Plot of the AUC values with 95% C.I for the NND, lacunarity, and succolarity at different distances from the ONH for retina which were dissected (A) 7 days, (B) 21 days, (C) 56 days, and (D) 84 days post model induction.

In Figure 7, a consistent trend is observed across all dissection times for lacunarity, succolarity, and NND. However, for the succolarity, it is observed that the spread of the AUC values is lower than that of the NND and lacunarity, and so the probability of observing a low AUC value for this measure is lower than that of the others. To further assess the performance of each measure at each dissection time, the AUC values were examined at different distances from the ONH. The results of which are displayed in Figure 8.

When examining retinas dissected after 7 days, these three measures (lacunarity, succolarity, and NND) consistently perform well in disease detection, with AUC values exceeding 0.7 throughout the central retina. This robust trend persists across different dissection times, underscoring the effectiveness of these measures in early disease detection.

## 5 Discussion

This study demonstrates that fractal characterizations of noisy and error‐prone point patterns can significantly improve the early detection of neurodegenerative changes in conditions such as glaucoma. By analyzing point patterns from established rodent models of glaucomatous neurodegeneration, we identified succolarity and lacunarity as superior metrics compared to traditional measures. These findings not only advance our understanding of spatial point patterns but also provide practical tools for clinical applications.

A key finding is that succolarity and lacunarity outperform FD in capturing subtle changes in RGC point patterns. While FD has been widely used in spatial analyses [26, 27, 28], its reliance on the box‐counting algorithm limits its sensitivity. Fractal dimension is estimated through a binary occupancy approach, where the space is partitioned into a grid of boxes and the presence or absence of at least one point within each box is recorded. This means that FD primarily captures whether spatial regions are occupied, rather than how points are distributed within those regions. In the context of retinal ganglion cell mosaics, where neurodegeneration occurs gradually through sparse and localized cell loss, this creates an important limitation. A box that remains partially populated will still be counted as occupied even if a substantial number of cells within it have been lost. As a result, early or moderate levels of degeneration may not meaningfully alter the scaling relationship that FD depends on, leading to a relative insensitivity to subtle structural change.

This reflects an estimator‐level limitation of the box‐counting approach rather than a failure of fractal theory itself. In contrast, metrics such as lacunarity and succolarity explicitly capture spatial heterogeneity and void structure, making them more responsive to changes in local density and spatial organization characteristic of early neurodegeneration. This method quantifies the presence of points within boxes but neglects their spatial distribution, which is critical for detecting early neurodegenerative changes [29]. Unlike FD, succolarity and lacunarity incorporate detailed information about the dispersion and gaps within point patterns, making them more effective for early‐stage disease detection. These findings address a notable gap in previous research, where FDs limitations were acknowledged but alternative metrics were not extensively explored in the context of glaucoma [26, 30].

Prior work has demonstrated that fractal dimension (FD) and lacunarity capture complementary aspects of spatial structure, and that their joint use can improve discrimination performance compared to either measure alone. In particular, Kilic and Abiyev [31] showed that combining FD and lacunarity enhances texture classification accuracy by jointly capturing global scaling behaviour and local heterogeneity. Similarly, Zaia et al. [32] reported that FD‐lacunarity analysis of trabecular bone in MRI provides improved structural characterization compared to single‐metric approaches.

Despite this established complementarity in other imaging domains, the combined use of FD and lacunarity has not been systematically explored in the context of glaucoma‐related retinal analysis. In this study, both metrics were evaluated independently as descriptors of spatial complexity in retinal structures. The observed relationship between FD and lacunarity suggests a degree of shared structural sensitivity, although they are not redundant and instead reflect different scales of organization. This raises the possibility that a combined or weighted integration of these features may further improve diagnostic performance. However, determining the optimal strategy for combining these measures, whether through linear weighting, multivariate modelling, or machine learning approaches, requires further investigation beyond the scope of the present study.

The robustness of succolarity and lacunarity to noise and sampling variability further underscores their clinical utility. Unlike nearest neighbor distance (NND), which can be distorted by noise, succolarity and lacunarity maintain their reliability even under challenging conditions. Simulation studies confirmed that these metrics remain stable when subjected to Gaussian noise, conditions that are often encountered in clinical data sets. This robustness reduces the need for repeated testing, potentiallysaving time and costs in clinical workflows while enhancing diagnostic reliability.

The implications of these findings extend beyond research to practical clinical applications. Succolarity and lacunarity offer a pathway for integrating advanced spatial analysis into routine diagnostics, particularly with the advent of imaging technologies such as Adaptive Optics Confocal Scanning Laser Ophthalmoscopy (AO‐cSLO) [9] and Detection of Apoptosing Retinal Cells (DARC) [32]. These modalities provide the high‐resolution imaging necessary to capture early neurodegenerative changes, making them ideal for validating these metrics in human studies. Furthermore, the stability of these measures in the face of noise and variability suggests they could be seamlessly incorporated into diagnostic workflows, reducing costs and improving efficiency.

Future directions for this work include validating these metrics in human retinal data sets, exploring their applicability to other neurodegenerative conditions such as Alzheimer's disease or Parkinson's disease, and optimizing them for real‐time clinical decision‐making. By scaling these tools for use in clinical environments, succolarity and lacunarity have the potential to revolutionize early diagnosis and treatment monitoring across a range of diseases.

This study establishes succolarity and lacunarity as reliable, early, and robust indicators of neurodegeneration in spatial point patterns. By addressing the limitations of traditional metrics and providing practical tools for implementation, we have laid the groundwork for innovative diagnostic approaches. These measures not only enhance the early detection of glaucoma but also hold promise for broader applications in neurodegenerative research and clinical practice, prioritizing early intervention and cost‐effective workflows.

## Funding

This work was supported by the Department for the Economy.

## Disclosure

The authors have nothing to report.

## Conflicts of Interest

The authors declare no conflicts of interest.

## Figures and Tables

**FIGURE B1:**
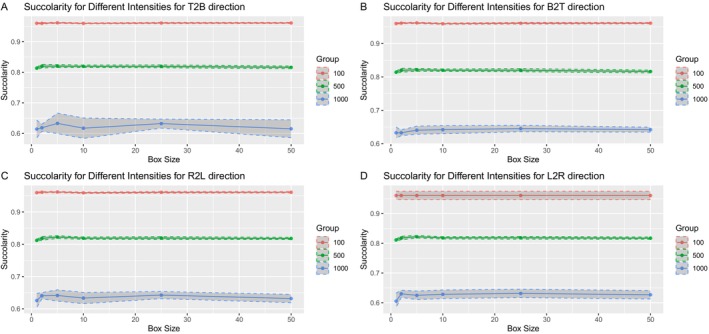
The succolarity for the different box lengths.

**FIGURE B2:**
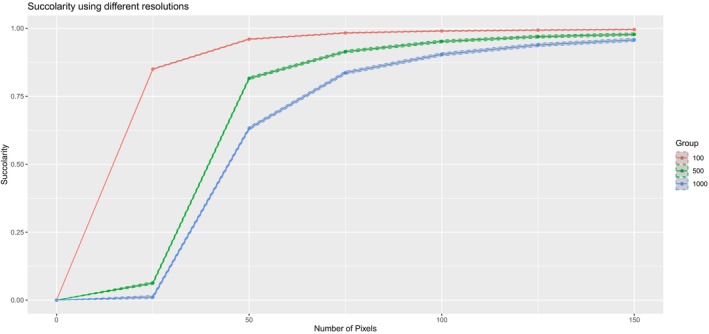
The succolarity for different resolution of images used.

## Data Availability

Code and datasets are available upon request. The code includes the parameter settings and enables lacunarity and succolarity to be computed over a range of settings.

## Appendix A Effects of Noise on Metrics Using Clustered and Inhibition Processes

In this appendix, we present the analysis examining the effects of noise on the measured metrics for clustered and inhibition point patterns. The Tables A1, A2, A3, A4 below summarise the results for both the clustered and inhibition processes.

**TABLE A1:** Values for each metric for different levels of positional noise using a clustered point pattern.

Noise Level	NND	NND C.I	FD	FD C.I	Lacunarity	Lacunarity C.I	Succolarity	Succolarity C.I
Baseline Pattern	0.03	[0.025,0.034]	1.64	[1.51,1.77]	0.95	[0.85, 1.02]	0.915	[0.899, 0.923]
0.01	0.032	[0.027,0.036]	1.65	[1.54,1.85]	0.91	[0.87, 0.99]	0.912	[0.893, 0.922]
0.05	0.041^a^	[0.035,0.044]	1.71	[1.62,1.85]	0.90	[0.83, 0.95]	0.908	[0.887, 0.920]
0.1	0.046^a^	[0.041,0.050]	1.73	[1.65,1.81]	0.88	[0.81, 0.93]	0.905	[0.891, 0.918]
0.2	0.049^a^	[0.043,0.054]	1.74	[1.67,1.81]	0.85	[0.78, 0.92]	0.913	[0.886, 0.921]
0.3	0.048^a^	[0.044,0.052]	1.78	[1.70,1.85]	0.89	[0.81, 0.93]	0.919	[0.901, 0.926]
0.4	0.052^a^	[0.048,0.055]	1.83^a^	[1.78,2]	0.91	[0.85, 0.98]	0.922	[0.902, 0.927]

^a^ Indicates significant difference from the baseline pattern.

**TABLE A2:** Values for each metric for different levels of positional noise using a point pattern generated by a inhibiton process.

Noise Level	NND	NND C.I	FD	FD C.I	Lacunarity	Lacunarity C.I	Succolarity	Succolarity C.I
Baseline Pattern	0.052	[0.045,0.054]	1.72	[1.61,1.95]	0.43	[0.35, 0.51]	0.885	[0.875, 0.893]
0.01	0.055	[0.045,0.058]	1.75	[1.63,1.98]	0.46	[0.35, 0.53]	0.881	[0.874, 0.894]
0.05	0.047	[0.043,0.052]	1.78	[1.67,2]	0.49	[0.38, 0.56]	0.885	[0.876, 0.891]
0.1	0.042^a^	[0.038,0.044]	1.83	[1.65,2]	0.52	[0.45, 0.59]	0.892	[0.882, 0.895]
0.2	0.042^a^	[0.037,0.044]	1.84	[1.69,2]	0.39	[0.34, 0.51]	0.890	[0.878, 0.897]
0.3	0.039^a^	[0.033,0.042]	1.87	[1.7,2]	0.45	[0.38, 0.53]	0.891	[0.882, 0.903]
0.4	0.041^a^	[0.036,0.044]	1.83	[1.66,2]	0.46	[0.37, 0.55]	0.898	[0.885, 0.909]

^a^ Indicates significant difference from the baseline pattern.

**TABLE A3:** Values for each metric for different levels of noise by introducing false positive and negatives using a point pattern generated by a clustered process.

Noise Level (False Positive, False Negative)	NND	NND C.I	FD	FD C.I	Lacunarity	Lacunarity C.I	Succolarity	Succolarity C.I
Baseline Pattern	0.033	[0.026,0.035]	1.65	[1.53,1.92]	0.99	[0.94, 1.05]	0.922	[0.910, 0.929]
(0.1,0.1)	0.037	[0.029,0.039]	1.66	[1.54,1.95]	1.02	[0.94, 1.06]	0.918	[0.904, 0.922]
(0.2,0.2)	0.043	[0.033,0.046]	1.68	[1.54,1.94]	1.04	[0.96, 1.07]	0.915	[0.901, 0.920]
(0.3, 0.3)	0.045^a^	[0.039,0.048]	1.71	[1.55,1.91]	1.01	[0.95, 1.04]	0.914	[0.901, 0.919]
(0.4, 0.4)	0.046^a^	[0.038,0.050]	1.69	[1.53,1.92]	0.97	[0.92, 1.03]	0.917	[0.907, 0.921]

^a^ Indicates significant difference from the baseline pattern.

**TABLE A4:** Values for each metric for different levels of noise by introducing false positive and negatives using a point pattern generated by a inhibition process.

Noise level (False positive, false negative)	NND	NND C.I	FD	FD C.I	Lacunarity	Lacunarity C.I	Succolarity	Succolarity C.I
Baseline Pattern	0.051	[0.044,0.054]	1.82	[1.74,1.95]	0.43	[0.34, 0.48]	0.882	[0.878, 0.886]
(0.1, 0.1)	0.053	[0.046, 0.057]	1.84	[1.75,1.94]	0.46	[0.38, 0.51]	0.883	[0.876, 0.886]
(0.2, 0.2)	0.054	[0.049, 0.058]	1.83	[1.73,1.94]	0.47	[0.39, 0.54]	0.886	[0.881, 0.889]
(0.3, 0.3)	0.059^a^	[0.055, 0.062]	1.85	[1.71,1.92]	0.47	[0.41, 0.52]	0.885	[0.881, 0.888]
(0.4, 0.4)	0.062^a^	[0.057, 0.065]	1.87	[1.73,1.93]	0.49	[0.43, 0.54]	0.881	[0.878, 0.887]

^a^ Indicates significant difference from the baseline pattern.

## Appendix B Resolution and Box Size Selection for Succolarity

In the calculation of succolarity, two factors need to be considered. First, how to choose the box length for calculating the occupation percentage, and second, how fine a resolution to use when converting the point pattern to a pixel image. To address the first question, a simulation study was conducted where a point pattern was generated from a homogeneous Poisson point process with intensities of 100, 500, and 1000. For each model, 1000 simulations were carried out, and the succolarity was calculated for each simulation, considering box lengths of 1, 2, 5, 10, 25, and 50 pixels. The resolution for each generated point pattern was kept constant at 50×50 pixels. The results, including the average succolarity for each box length and the 95% confidence interval, are shown for each of the four directions in Figure B1.

The results indicate that there is little variation in the value of succolarity across different intensities. Even at lower intensities of 100 and 500, there is almost no variation in value. In the extreme case where the intensity is 1000, there is still very little change in the value. Specifically, the maximum difference between the largest and smallest values within the entire simulation for each of the four directions is only 0.06, and there is no significant difference between the values. Therefore, the choice of box size has no influence on the overall value of succolarity. The only restriction is that it must be chosen as a factor of the resolution used. The second question to be addressed in this section is the resolution of the image representing the point pattern. In some cases, the point pattern may contain covariate information, known as marks. These marks may represent the diameter of a tree or the area of a cell, for example. In such cases, this information should guide the choice of resolution, aiming for the fraction of pixels in the resulting image representing points within the entire study region to be approximately equal to the fraction of the areacovered by the marked points within the pattern. However, when the pattern lacks marks or covariate information, Auntunes et al. [13] suggest that the value of the measure under consideration, namely, succolarity, should be calculated at multiple resolutions. The chosen resolution should be the one where the curve exhibits a “knee,” meaning it has the largest magnitude difference between two points. Figure B2 illustrates a simulation for calculating the resolution in the case of a homogeneous Poisson process with intensities of 100, 500, and 1000. Again, 1000 simulations were performed for each process, and a box length of 5 was chosen for succolarity, as the results in Figure B1 demonstrate that the box length does not affect succolarity.

Applying the technique from Antunes et al. [33] to Figure B2 shows that for a homogeneous Poisson process of intensity 100 the resolution to be used should be 50×50 and for processes with intensity of 500 and 1000 the resolution should be 75×75 for both.
